# Effects of affective picture viewing on postural control

**DOI:** 10.1186/1471-2202-8-83

**Published:** 2007-10-04

**Authors:** John F Stins, Peter J Beek

**Affiliations:** 1 Research Institute MOVE, Faculty of Human Movement Sciences, VU University Amsterdam, van der Boechorststraat 9, 1081 BT, Amsterdam, The Netherlands

## Abstract

**Background:**

Emotion theory holds that unpleasant events prime withdrawal actions, whereas pleasant events prime approach actions. Recent studies have suggested that passive viewing of emotion eliciting images results in postural adjustments, which become manifest as changes in body center of pressure (COP) trajectories. From those studies it appears that posture is modulated most when viewing pictures with negative valence. The present experiment was conducted to test the hypothesis that pictures with negative valence have a greater impact on postural control than neutral or positive ones. Thirty-four healthy subjects passively viewed a series of emotion eliciting images, while standing either in a bipedal or unipedal stance on a force plate. The images were adopted from the International Affective Picture System (IAPS). We analysed mean and variability of the COP and the length of the associated sway path as a function of emotion.

**Results:**

The mean position of the COP was unaffected by emotion, but unipedal stance resulted in overall greater body sway than bipedal stance. We found a modest effect of emotion on COP: viewing pictures of mutilation resulted in a smaller sway path, but only in unipedal stance. We obtained valence and arousal ratings of the images with an independent sample of viewers. These subjects rated the unpleasant images as significantly less pleasant than neutral images, and the pleasant images as significantly more pleasant than neutral images. However, the subjects rated the images as overall less pleasant and less arousing than viewers in a closely comparable American study, pointing to unknown differences in viewer characteristics.

**Conclusion:**

Overall, viewing emotion eliciting images had little effect on body sway. Our finding of a reduction in sway path length when viewing pictures of mutilation was indicative of a freezing strategy, i.e. fear bradycardia. The results are consistent with current knowledge about the neuroanatomical organization of the emotion system and the neural control of behavior.

## Background

Recently there has been an upsurge of interest in the question to what extent the human motor control system is influenced by the emotional state of the actor. Several cortical and subcortical loops have been identified that provide an interface between the emotion system and the motor control system. As an example, limbic structures such as the anterior cingulate cortex and the orbitofrontal cortex receive input from the amygdala, and send projections to the basal ganglia via the so called limbic loop, also known as the anterior cingulate basal ganglia-thalamocortical circuit [1,2]. The ventral striatum also receives direct input from limbic structures such as the hippocampus, amygdala, and entorhinal cortices [3]. Thus, the basal ganglia are likely involved not only in involuntary bodily movements, such as gait and posture, but also in the bodily expression of emotions. As another example, the limbic association cortex, which includes the orbitofrontal cortex, the cingulate cortex and the parahippocampal area receives projections from the higher-order sensory areas and from limbic areas, and can affect emotion-mediated motor planning via its projections to the prefrontal cortex [3]. Despite neuroanatomical evidence regarding the interface between limbic and motor control structures, current knowledge about the precise interrelations between these systems is still limited and, as far as we know, motor control models with an explicit affective component remain to be developed.

The interrelatedness between the affective system and the motor control system likely serves an evolutionary purpose. Darwin [4] already argued that emotions are adaptive, in the sense that they prime a behavioral response pattern that is appropriate to deal with the environmental event that triggered the emotion. In an attempt to classify the wide variety of emotions, theorists in the field of behavioral neuroscience and psychology tend to agree that one overarching component of each emotion is its hedonic valence, i.e., the experienced pleasantness or unpleasantness. Within this framework, emotions either have an appetitive (approach) motivational component or a defensive (withdrawal) component (e.g., [5]). For present purposes we focus on unpleasant emotions such as fear and disgust, because they tend to be processed via a rapid subcortical projection, preparing the animal for quick and appropriate reactions (e.g. [6]). In this respect an important neural structure is the amygdala which, via projections to the periaqueductal gray (PAG), can trigger emotional response output, such as a fight-or-flight response or freezing behavior [7]. Furthermore, the amygdala can induce an increase in autonomic activity such as an increase in heart rate via the lateral hypothalamus.

Much of our current knowledge about interactions between the emotion system and motor system comes from animal research, such as aversive conditioning, but relatively little is known about how these systems interact to affect the behavior of healthy humans. A few studies have shown that emotions, especially negative ones, can prime adaptive behavioral responses, such as approach-avoidance behavior or freezing behavior. These responses become manifest as subtle changes in body posture, which is the focus of the present study.

Three recent studies adopted a behavioral paradigm whereby participants were asked to passively view a set of emotionally charged images that were adopted from the International Affective Picture System (IAPS; [8]). The pictures were shown while participants were standing on a force plate, and resultant changes in the body center of pressure (COP) were taken as a measure of the bodily preparedness to engage in adaptive emotional behavior. First, a study by Hillman et al. [9] found postural adjustments when viewing affective pictures, especially when viewing unpleasant images depicting scenes of attack or mutilation. More specifically, in response to unpleasant images the COP of males displayed small anterior (forward) postural adjustments, suggesting an approach action tendency. In contrast, the COP of females displayed posterior adjustments, suggesting a defensive (flight) tendency. Thus, emotionally charged images caused unintentional postural adjustments, and this effect was modulated by the gender of the viewers. Second, a study by Azevedo et al. [10] examined in more detail the effects of pictures of mutilation on posture and heart rate. The pictures were supposed to induce an emotion of disgust with the viewers, and postural responses were compared to neutral pictures (objects) and pleasant/arousing pictures (sport scenes). In males, this class of pictures resulted in a significant overall reduction of body sway, as evidenced by a reduction in the standard deviation of the COP trajectory. Furthermore, the unpleasant images caused a significant heart rate deceleration. These postural and physiological responses were strongly suggestive of activation of the defensive system, resulting in bodily freezing and fear bradycardia. Finally, similar findings were reported by Facchinetti [11], who found a reduction in postural mobility and an increase in mean power frequency of the COP signal in response to pictures of mutilation (but also in response to affiliative pictures, such as babies and families), compared to blocks of neutral stimuli. Furthermore, pictures of mutilation resulted in a significant heart rate deceleration. These findings were also consistent with freezing behavior in response to a threatening context.

The aim of the present study was to examine how postural sway is influenced when viewers are confronted with emotion eliciting pictures. Based on the studies described in the preceding section we predicted that especially pictures with negative emotional content have a discernible influence on postural sway. More specifically, we predicted that unpleasant images will result in small backward displacements of the COP, indicative of withdrawal behavior. In addition, unpleasant images depicting disgusting scenes were expected to lead to smaller COP excursions (freezing) than with other images. We employed a paradigm similar to the one used by [9] but with two major changes. First, we modified the order of presentation of the images. In previous studies using IAPS images [9-11] all images within an emotion category were presented in a blocked order. This procedure could have biased the participants, in that they always knew within a block what kind of emotional experience the stimulus was expected to induce. Relatedly, the blocking procedure could cause the effects of emotion to accrue over time, due to increased sensitivity to the images. This makes it difficult to attribute the observed postural adjustments to individual stimuli because subjects could somehow engage in anticipatory bodily behavior throughout a block of stimuli. Based on these considerations we decided to administer the stimuli in a random order. Although this design feature is likely to lead to smaller overall effects (see also [12]), it produces a more valid measure of emotion. Second, we included an additional postural manipulation. The studies described above involved 'normal' standing, that is, standing on two legs on a rigid support surface. It is known that by introducing an additional challenge to posture such as standing on foam or standing with eyes closed, maintaining balance becomes more difficult (possibly requiring more attentional 'resources' [13]) which, in turn, may cause a reduction in postural stability and greater sway. We therefore hypothesized that the putative effects of emotion on balance would be amplified when the balance system is challenged. To this end, we asked participants to view the affective pictures while standing on two legs (bipedal stance) and while standing on one leg (unipedal stance; see also[14]). We expected that the effects of emotion on posture would be larger during unipedal stance than bipedal stance.

## Results

All participants completed the experiment. Fourteen trials (out of a total of 2958) had to be discarded due to loss of balance in the unipedal condition.

### Valence and arousal ratings

Using pair-wise t-tests we found that pleasant images were rated as significantly more pleasant than neutral images (5.3 vs. 4.0; t(12) = 6.54; p < .001), and that unpleasant images were rated as significantly less pleasant than neutral images (1.8 vs. 4.0; t(12) = 14.29; p < .001). We also found that pleasant images were rated as significantly more arousing than neutral images (3.5 vs. 1.2; t(12) = 8.62; p < .001), and also that unpleasant images were rated as significantly more arousing than neutral images (4.8 vs. 1.2; t(12) = 11.80; p < .001).

The transformed valence scores of Hillman et al. [9] were 6.2, 4.3, and 1.8 for pleasant, neutral, and unpleasant pictures, respectively. In two instances the Hillman et al. [9] valence scores were significantly higher than in our sample (pleasant: t(12) = 3.75, p < .01; neutral: t(12) = 3.33, p < .01). For the unpleasant images the difference was not significant. The transformed arousal scores of Hillman et al. [9] were 4.7, 2.8, and 6.3 for pleasant, neutral, and unpleasant pictures, respectively. In all three instances these scores were significantly higher than in our sample (pleasant: t(12) = 3.63, p < .01; neutral: t(12) = 5.71, p < .001; unpleasant: t(12) = 4.84, p < .001). In sum, our sample of Dutch viewers rated the images as overall less pleasant and less arousing than their American counterparts.

### Postural effects

The ANOVA performed on COP-AP only revealed a main effect of emotion; F(2, 64) = 3.771; p < .05. Both the neutral and unpleasant pictures resulted in a modest 1-mm forward displacement of the COP, whereas the COP displacement with pleasant pictures was virtually zero. No other effects were significant.

The ANOVA performed on SD [COP] only revealed a main effect of stance: F(1, 32) = 155.221; p < .001. Unipedal stance was more variable than bipedal stance (5 mm vs. 2 mm).

**Figure 1:**
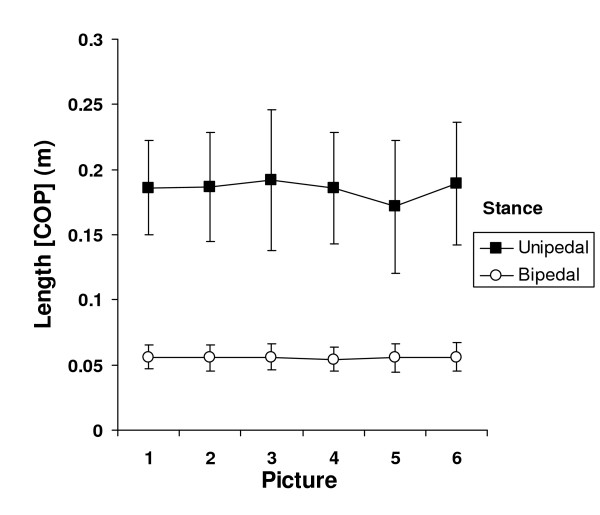
Means (+/- 1 standard deviation) of sway path length as a function of stance (unipedal/bipedal) and picture. 1 = faces; 2 = household objects; 3 = erotica; 4 = family scenes; 5 = mutilation; 6 = attack.

**Figure 2:**
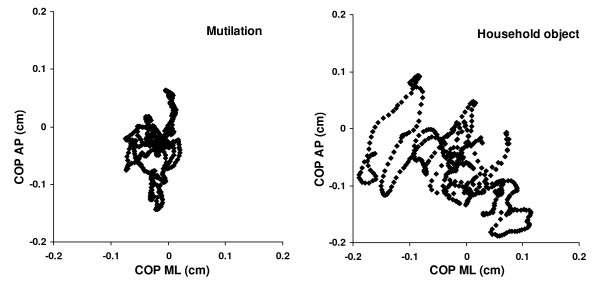
Representative time series of the COP from one participant in unipedal stance while watching a scene depicting mutilation (left) and a picture of a household object (right). AP is anterior-posterior; ML is medio-lateral.

In a similar vein, the ANOVA performed on Length [COP] also revealed a main effect of stance; F(1, 32) = 838.121; p < .001. The length of the sway path during unipedal stance was greater than during bipedal stance (18.7 cm vs. 5.6 cm). Furthermore, the stance × picture interaction was significant; F(5, 160) = 3.763, p < .01. The means are shown in Figure 1. Inspection of the means revealed that sway path was shorter for pictures of mutilation compared to the other pictures but only with a unipedal stance (17.2 cm vs. 18.8 cm; Figure 2).

Pairwise t-tests between pictures of mutilation and all other picture categories revealed in all instances in the unipedal condition a significant (p < .05, uncorrected) difference.

## Discussion

The aim of this study was to test whether the limbic-motor interaction would show up as behavioral adjustments of body posture in response to emotion eliciting images. Based on neuroanatomical knowledge and on previous studies using a similar paradigm we predicted that especially unpleasant emotions would have a discernible effect on body sway. The results revealed only modest effects of emotion on the COP. We were unable to replicate the findings of Hillman et al. [9]. However, we were able to partially replicate the results of previous studies [10,11], in that our pictures of mutilation resulted in a shorter total sway path compared to the other pictures, at least in the unipedal stance condition. This finding is consistent with the hypothesis that perception of threat results in some degree of bodily "freezing", also known as fear bradycardia, or "attentive immobility" [15], which can be described as a state of hypervigilance. This state is characterized by heart rate deceleration and a concommittant increase in attentional processing [16].

There may be several reasons why the observed effects of emotion on COP changes were reduced compared to previous studies. First, it could be the case that our procedure involving randomization of the stimuli somehow 'washed out' the emotion inducing effects. The rapid and unpredictable succession of pleasant, unpleasant, and neutral images may simply not have left enough room for clear and powerful emotions to become manifest. However, we believe this is unlikely because other studies have successfully adopted randomization in this context: Coombes et al. [17] induced an emotion-movement interaction using complete randomization of IAPS images (neutral, pleasant, and unpleasant), and it was found that the magnitude of sustained contraction of hand muscles was greater with unpleasant images than with the other categories. Furthermore, Bradley et al. [18] obtained reliable psychophysiological changes as a function of picture category, again employing a complete random order of IAPS images. Thus, even when emotion eliciting images are presented in a random order they can still yield clearly measurable effects.

Second, it could be that the adopted stimuli were not suitable for our audience of Dutch viewers. After the experiment some participants remarked that some of the pictures displaying scenes of attack and of erotica were awkward, and that they rarely resulted in strong emotional feelings. The IAPS was validated with American viewers, and cultural differences render it possible that the IAPS is more suitable for an American audience than a European one. Our assessment of the same IAPS-images with an independent group of Dutch viewers yielded clear effects of valence and arousal in the expected direction. However, the overall valence scores and arousal scores were significantly lower than the scores reported in [9], who used the same stimuli. Thus, it could be that the images only resulted in weak emotional feelings with our group of subjects, and hence only modest postural adjustments.

Third, it could be that passive viewing of images is only weakly coupled to posture. A probably more effective way to probe the emotion-posture system would be to induce an emotional state that is somehow relevant to the postural control system. For example, anxiety can be successfully introduced by having participants maintain balance while standing close to an edge, which then primes the balance system for fear of falling. This manipulation was introduced by Carpenter et al. [19] who found that standing on a surface height resulted in a posterior displacement, i.e., away from the edge, with values that were about twice as large as those reported by Hillman et al. [9]. Furthermore they [19] found that increasing the surface height caused participants to adopt a freezing strategy, as exemplified by an increase in mean power frequency and a concurrent decrease in amplitude variability of the COP.

## Conclusion

We only found modest effects of affective picture viewing on COP changes. Body sway was only affected when balance was challenged and when subjects viewed pictures with disgusting content. This result is consistent with the hypothesis that neural structures responsible for negative affect can exert their influence on structures responsible for adaptive behavioral actions.

## Methods

Thirty-four participants (17 males, 17 females), aged between 18 and 30 years participated in this study. The participants had no known visual or motor impairments. The study was approved by the local Ethics Committtee. All participants signed an informed consent form.

Posturographic data were recorded at 100 Hz using a Kistler forceplate (Kistler 9865B B; Kistler Instrumente AG Winterthur, Switzerland). From those data the COP was calculated in the anterior-posterior (AP) and medio-lateral (ML) direction.

The participants viewed a total of 87 pictures (29 pleasant, 29 neutral, 29 unpleasant) from the International Affective Picture System [8]. Our set included the 60 IAPS images used by [9]. The pleasant pictures included family scenes and erotic scenes; the neutral pictures included pictures of faces and household objects; the unpleasant pictures included scenes of attack by humans or animals and scenes of mutilation. The complete list of pictures we used from the IAPS is shown in the Appendix.

Valence and arousal ratings of each image were obtained from a separate group of 13 subjects (6 males; 7 females; mean age 21.9 years; SD = 3.2 years) following the instruction manual of the IAPS. For both dimensions a 9-point rating scale was used (1 – 9) and subjects were asked to rate their experienced level of valence (V) and arousal (A) associated with each image. High (low) values of V denote high (low) levels of pleasantness, and high (low) values of A denote high (low) levels of experienced arousal. Using a t-test we compared valence and arousal ratings as a function of picture category.

We also compared our valence and arousal scores to the scores obtained with the thirty-six subjects in the Hillman et al. [9] sudy, in order to directly compare the results of our study to theirs. In our study the ratings were obtained on a 9-point scale, whereas in their [9] study the ratings were obtained on a 21-point scale (0 to 20). Both scales make use of the same self-assessment manikin (SAM; [8]). In this system, a graphic figure displaying various facial expressions is used. For the valence ratings, SAM ranges from a smiling happy figure to a frowning unhappy figure. For the arousal ratings, SAM ranges from an excited, wide-eyed figure to a relaxed sleepy figure. Both scales consists of 5 SAM figures, and in our study subjects could (using a paper and pencil) put a mark either on the figure itself, or on one of the four intermediate positions, thus resulting in a 9-point scale. In the computerized version of Hillman et al. [9] the same 5 SAM figures were used, but between each figure there were four points that could be selected, thus resulting in a 21-point scale. Given that effectively the same measurement tool was used, although our scale had lower discriminative power, we felt it legitimate to linearly transform the Hillman et al. [9] scores to our range. We first multiplied the values in Hillman et al.'s [9] Table 1 (averaged over males and females) by 9/21, and we then compared these transformed values to our values using a 2-tailed t-test.

Participants stood in the middle of the force plate with their arms hanging relaxed alongside their body. In front of the participants at a distance of 1.2 m was a 17-inch monitor, positioned at eye height, onto which the stimuli were shown in full-screen mode. The experiment took place in a darkened room, resulting in clear and vivid images. Participants were instructed to maintain a comfortable and upright stance, and to simultaneously watch the images that were shown on the screen.

In the bipedal stance condition participants stood barefoot on the plate with their feet slightly pointing outward. The bidepal condition started with a 60-s baseline measurement, during which no images were shown. These data were not analyzed further. Next, 6 sets of images were shown. Each set consisted of 10 IAPS images that were shown in succession. Each image was shown for a duration of 5 s, and was preceded by a 2-s black screen. The COP trajectories during the presentation of the black screen were used later for baseline correction. The images were shown in a random order. After each set of images participants received a 30-s break during which they could stretch their legs and briefly leave the force plate if they desired. In addition, participants had to answer three questions concerning the images shown (e.g., "did you see a green snake?") with a "yes" or a "no". Subjects did not know in advance which questions they would receive. The purpose of the questions was to ensure that the images were actually attended to during the experiment.

In the unipedal stance condition participants stood on their preferred leg (based on self report) with the other leg raised slightly above the ground. The unipedal condition was identical to the bipedal condition, but with fewer stimuli in order to prevent fatigue or discomfort due to the unnatural stance. In this condition 3 sets of 9 stimuli were presented bringing the total number of stimuli (bipedal plus unipedal) to 87. The bidepal condition always preceded the unipedal condition. Performance was monitored by an experimenter, who could abort a trial when a participant lost his or her balance, which happened only rarely.

Prior to data reduction we baseline corrected the 5-s COP time series of each trial by subtracting the mean value of the COP in the anterior-posterior direction (COP-AP) of the 2-s black screen that preceded the stimulus. This was done to obtain the 'true' change in mean COP-AP as a function of stimulus, unaffected by subtle variations in initial stance position within and between participants.

For each trial we calculated the following values: 1) the mean COP in the anterior-posterior direction (COP-AP), which is an index of the extent to which a participant is leaning in the anterior or posterior direction during a trial, 2) the standard deviation of the COP in AP direction (SD [COP]), which measures stability of the upright stance in the sagittal plane, and 3) the length of the sway path of the COP in the horizontal plane (Length [COP]), which is an index of the total amount of body sway. These values were submitted to a 2 (gender: male/female) × 2 (stance; bipedal/unipedal) × 3 (neutral, pleasant, and unpleasant images) repeated measures analysis of variance (ANOVA). In addition, we analyzed the effects of picture category on posture, because previous work [10] showed that certain picture categories (most notably pictures of mutilation) give rise to a distinctive COP pattern. We therefore analyzed sway path length as a function of gender, stance, and picture (household objects, faces, erotica, family scenes, mutilation, attack).

## Abbreviations

COP: center of pressure

PAG: periaqueductal gray

IAPS: International Affective Picture System

ANOVA: analysis of variance

AP: anterior-posterior

ML: medio-lateral

SD: standard deviation

SAM: self-assessment manikin

## Authors' contributions

JFS and PJB conceived the study's paradigm. JFS evaluated the data and results, and wrote the initial draft of the manuscript. PJB was further involved in revising the manuscript. Both authors read and approved the final manuscript.

## Appendix

A list of pictures from the IAPS that were used in the experiment. Means and ± S.D.s of the valence (V) and arousal (A) ratings, obtained with a separate group of viewers, of each category are also shown in parentheses. High (low) values of V denote high (low) levels of pleasantness, and high (low) values of A denote high (low) levels of experienced arousal.

Neutral/faces (V: 3.9 ± 0.5, A: 1.8 ± 1.2): 2190, 2200, 2210, 2214, 2215, 2221, 2270, 2271, 2280, 2383, 2440, 2512, 2516, 2570. Neutral/household objects (V: 4.1 ± 0.3, A: 0.6 ± 1.0): 7000, 7002, 7004, 7009, 7010, 7025, 7030, 7035, 7050, 7052, 7060, 7090, 7150, 7175, 7211. Pleasant/erotica (V: 4.8 ± 1.1, A: 3.8 ± 1.6): 4002, 4180, 4210, 4232, 4250, 4255, 4460, 4510, 4520, 4531, 4572, 4607, 4608, 4652, 4659, 4670, 4800. Pleasant/family (V: 6.0 ± 0.7, A: 3.0 ± 1.0):, 2057, 2070, 2080, 2165, 2260, 2311, 2340, 2341, 2360, 2387, 2391, 2660. Unpleasant/mutilation (V: 0.6 ± 0.5, A: 5.9 ± 1.2): 3000, 3010, 3053, 3060, 3064, 3080, 3100, 3110, 3130, 3150, 3170. Unpleasant/fear (V: 2.5 ± 0.6, A: 4.2 ± 1.1): 1050, 1120, 1200, 1201, 1300, 1301, 1930, 1932, 3022, 3550, 6190, 6230, 6250, 6260, 6300, 6313, 6350, 6560.

## Competing interests

The author(s) declares that there are no competing interests.

## References

[B1] Alexander GE, Crutcher MD (1990). Functional architecture of basal ganglia circuits: neural substrates of parallel processing. Trends Neurosci.

[B2] Alexander GE, Crutcher MD, DeLong MR (1990). Basal ganglia-thalamocortical circuits: Parallel substrates for motor, oculomotor, "prefrontal" and "limbic" functions. Prog Brain Res.

[B3] Kandel ER, Schwartz JH, Jessell TM (2000). Principles of neuroscience.

[B4] Darwin C (1872). The expression of emotion in man and animal.

[B5] Lang PJ, Bradley MM, Cuthbert BN (1998). Emotion, motivation, and anxiety: brain mechanisms and psychophysiology. Biol Psychiat.

[B6] LeDoux JE (1996). The emotional brain: The mysterious underpinnings of emotional life.

[B7] Amorapanth P, Nader K, LeDoux JE (1999). Lesions of periaqueductal gray dissociate conditioned freezing from conditioned suppression behavior in rats. Learn Mem.

[B8] Lang PJ, Bradley MM, Cuthbert BN (2005). International affective picture system (IAPS): Affective ratings of pictures and instruction manual. Technical Report A-6.

[B9] Hillman CH, Rosengren KS, Smith DP (2004). Emotion and motivated behavior: postural adjustments to affective picture viewing. Biol Psychol.

[B10] Azevedo TM, Volchan E, Imbiriba LA, Rodrigues EC, Oliveira JM, Oliveira LF, Lutterbach LG, Vargas CD (2005). A freezing-like posture to pictures of mutilation. Psychophysiology.

[B11] Facchinetti LD, Imbiriba LA, Azevedo TM, Vargas CD, Volchan E (2006). Postural modulation induced by pictures depicting prosocial or dangerous contexts. Neurosci Lett.

[B12] Stins JF, van Leeuwen WM, de Geus EJ (2005). The multi-source interference task: the effect of randomization. J Clin Exp Neuropsychol.

[B13] Donker S, Roerdink M, Greven A, Beek PJ (2007). Regularity of center-of-pressure trajectories depends on the amount of attentioninvested in postural control. Exp Brain Res.

[B14] Vuillerme N, Nougier V (2004). Attentional demand for regulating postural sway: the effect of expertise in gymnastics. Brain Res Bull.

[B15] Marks IM (1987). Fears, phobias and rituals.

[B16] Sanchez-Navarro JP, Martinez-Selva JM, Roman F (2006). Uncovering the relationship between defence and orienting in emotion: Cardiac reactivity to unpleasant pictures. Int J Psychophysiol.

[B17] Coombes SA, Caraugh JH, Janelle CM (2006). Emotion and movement: Activation of defensive circuitry alters the magnitude of a sustained muscle contraction. Neurosci Lett.

[B18] Bradley MM, Codispoti M, Cuthbert BN, Lang PJ (2001). Emotion and motivation I: defensive and appetitive reactions in picture processing. Emotion.

[B19] Carpenter MG, Frank JS, Silcher CP (1999). Surface height effects on postural control: A hypothesis for a stiffness strategy for stance. J Vestib Res.

